# Increased salt tolerance in *Zymomonas mobilis* strain generated by adaptative evolution

**DOI:** 10.1186/s12934-020-01406-0

**Published:** 2020-07-20

**Authors:** Katsuya Fuchino, Per Bruheim

**Affiliations:** Department of Biotechnology and Food Science, Norwegian University of Science and Technology, Trondheim, Norway

## Abstract

**Background:**

Ethanologenic alphaproteobacterium *Zymomonas mobilis* has been acknowledged as a promising biofuel producer. There have been numerous efforts to engineer this species applicable for an industrial-scale bioethanol production. Although *Z. mobilis* is robustly resilient to certain abiotic stress such as ethanol, the species is known to be sensitive to saline stress at a mild concentration, which hampers its industrial use as an efficient biocatalyst. To overcome this issue, we implemented a laboratory adaptive evolution approach to obtain salt tolerant *Z. mobilis* strain.

**Results:**

During an adaptive evolution, we biased selection by cell morphology to exclude stressed cells. The evolved strains significantly improved growth and ethanol production in the medium supplemented with 0.225 M NaCl. Furthermore, comparative metabolomics revealed that the evolved strains did not accumulate prototypical osmolytes, such as proline, to counter the stress during their growth. The sequenced genomes of the studied strains suggest that the disruption of ZZ6_1149 encoding carboxyl-terminal protease was likely responsible for the improved phenotype.

**Conclusions:**

The present work successfully generated strains able to grow and ferment glucose under the saline condition that severely perturbs parental strain physiology. Our approach to generate strains, cell shape-based diagnosis and selection, might be applicable to other kinds of strain engineering in *Z. mobilis*.

## Background

Biofuel is a renewable clean energy source derived from biomass. With increasing environmental concerns about the use of fossil-dependent fuels, development of sustainable biofuel-refinery has lately attracted public attention. Currently, the baker’s yeast *Saccharomyces cerevisiae* is regarded as the best promising bioethanol producer [1]. However, an alternative candidate, facultative anaerobic alphaproteobacterium *Zymomonas mobilis* is an efficient ethanol producer exhibiting several attractive physiological features surpassing *S. cerevisiae*, such as high specific rate of sugar uptake, relatively small genome size, low biomass production, a capacity to fix nitrogen gas [2–5], and superior ethanol productivity [4, 6]. Its efficient homo-ethanol fermentation, mediated by the Entner-Doudoroff (ED) pathway where up to 50% of total cellular soluble protein is involved as the catalytic enzymes, coupled to active pyruvate carboxylase and two alcohol dehydrogenases, catabolizes simple sugars efficiently and produces ethanol nearly at the theoretical maximum yield in *Z. mobilis* [4]. The potential of *Z. mobilis* culture in an industrial use is not strictly restricted in converting sugars to ethanol. It has been emerging as a producer of different high-value chemicals [7–9].

To expand its potential as a biocatalyst, increasing stress tolerance is critical in actualizing *Z. mobilis* based biorefinery. *Z. mobilis* cells are intrinsically tolerant of very high concentration of ethanol (> 10%, v/v) and broad pH range (3.5–7.5) [10]. However, the ubiquitous inorganic salt sodium chloride inhibits *Z. mobilis* growth at a mild level [10, 11]. Addition of 10 g/L NaCl in the complex growth medium, a concentration commonly used in bacterial growth medium, significantly perturbed the growth and ethanol production of *Z. mobilis* [11]. *Z. mobilis* also exhibits abnormal cell shape under the salt condition, by elongating rod/oval shape and forming long filamentous structure with a bulged swollen pole [11]. Considering that *Z. mobilis* grows well in the presence of high amounts of glucose [7], osmotic pressure per se may not be the sole cause, but ionic disturbance by salt likely attributes to the poor growth.

Inorganic ions are potential inhibitors in a lignocellulosic hydrolysate, an environmentally and economically appealing substrate for biofuel production. Also, the common salt NaCl may be found in different ranges of renewable industrial feedstocks [12]. De-salination before fermentation is not favored due to its high costs. Thus, *Z. mobilis* strains that can grow and ferment under salt condition are in demand. Nevertheless, only few studies have tacked this problem so far [13, 14]. Previously, a systematic approach introducing transposon mutations in *Z. mobilis* identified that a mutation in *himA* increased tolerance to the saline stress [13]. Although the mechanism of action was not clarified, the *himA* mutant strain exhibited improved growth and ethanol production under the saline condition. The Na^+^/H^+^ antiporter in strain ZM4 was also identified as an important transporter in the sodium ionic stress [14]. The strains obtained from these works showed promising improvements, yet, to a mild degree or under limited conditions. Interestingly, it was suggested the potential biological significance of respiratory chain—enigmatic, low-energy coupled aerobic respiration in *Z. mobilis* might be important for maintaining a low reduced/oxidized form of the co-factor Nicotinamide Adenine Dinucleotide (NAD) upon saline stress [15].

The general bacterial stress response to external high osmolality is to accumulate low molecular mass compatible solutes, through transporting and biosynthesis [16]. This type of response in *Z. mobilis* was previously reported, showing a relative increase of several metabolites under saline conditions [17]. On the other hand, Kohler et al. reported that *Z. mobilis* genome is missing most of the loci encoding compatible solute transport proteins such as the Kdp complex and BetS, implying that the transporting mechanism is not involved in this species to encounter saline stress [18]. In addition, accumulation of sorbitol was suggested to be important in osmotic stress response in *Z. mobilis* [19], however, this regulation was not confirmed in another study [15]. Taken together, these studies suggest that de novo synthesis of molecules appear to be critical for the adaptation in *Z. mobilis*.

For improving *Z. mobilis* cells against external abiotic stress, several studies adopted top-down or forward approaches. These includes an error-prone PCR based mutagenesis [20], an adaptive laboratory evolution method [21–23], genome shuffling [24] and a transposon approach [13].

In the present study, we improvised an adaptive laboratory evolution approach to generate saline resilient strains. The obtained strains successfully improved cellular growth and ethanol production under saline conditions. Furthermore, we examined the promising strains by quantitative metabolomics and whole genome sequencing, in order to understand what mechanism might be responsible for the salt tolerant phenotype.

## Results and discussion

### Adaptive laboratory evolution to generate salt-resilient strain

**Fig. 1:**
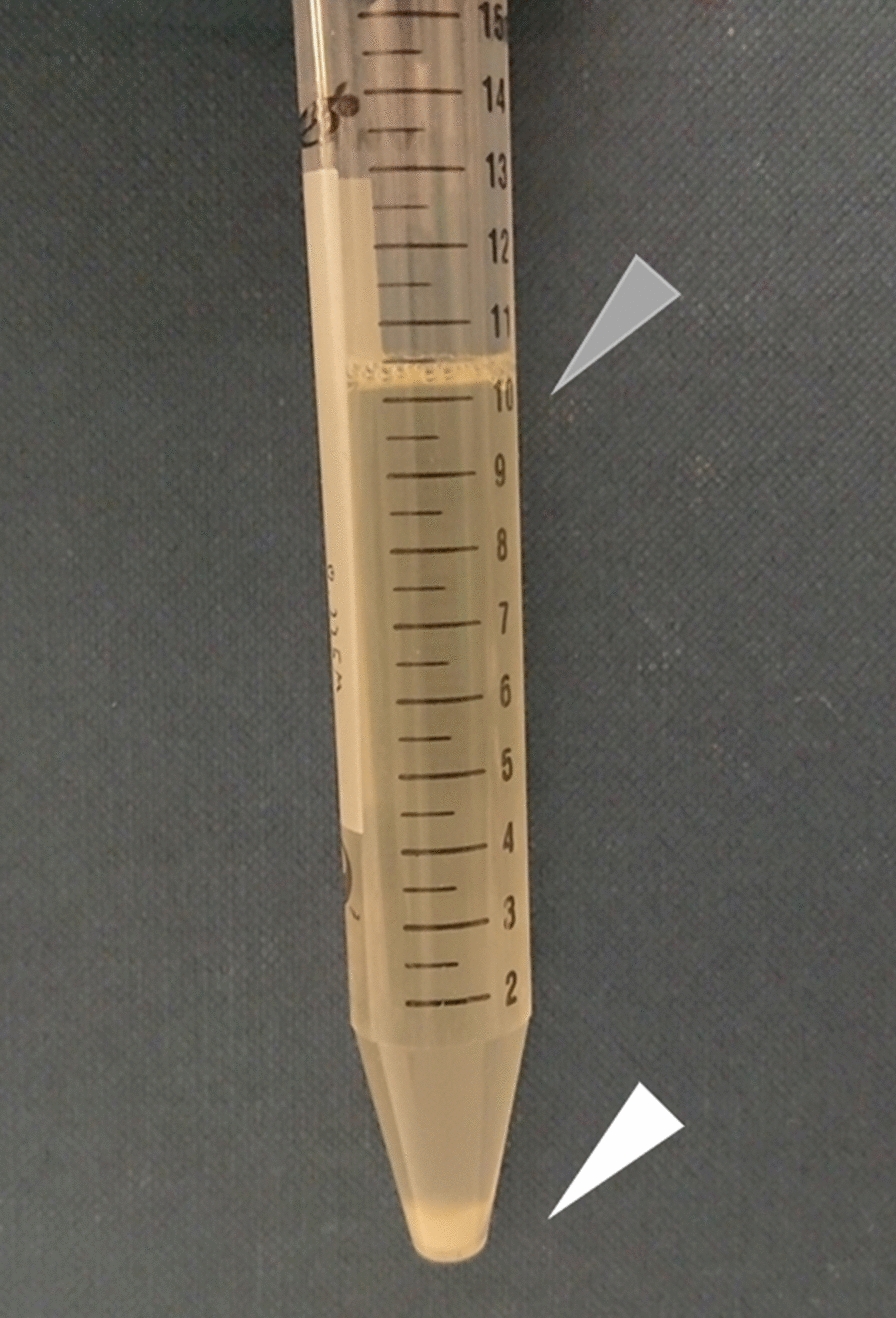
Morphology-biased serial transfer for generation of salt resilient *Z. mobilis* strains. The picture shows the fully grown Zm6 culture in the complex medium supplemented with NaCl 0.225 M. The sediments of Zm6 *Z. mobilis* cells at the bottom of culture (pointed by a white arrowhead), constituting clumps of bulged/filamented cells, were observed under a phase contrast microscope. In the upper layer of the medium (pointed by a gray arrowhead), shorter filamented cells resided, and this part of culture was transferred as an inoculum for next round of evolution

To generate *Z. mobilis* strains that can grow and ferment under saline stress conditions, we adopted an adaptive laboratory evolution strategy. The approach was previously employed by Wang et al., however the *Z. mobilis* culture with excessive NaCl concentration was not viable for long term due to a toxic effect, and the approach was not successful in generating resilient strain [13]. Therefore, we introduced a bias in the serial transfer to direct evolution preferable to our goal (Fig. 1).

**Fig. 2:**
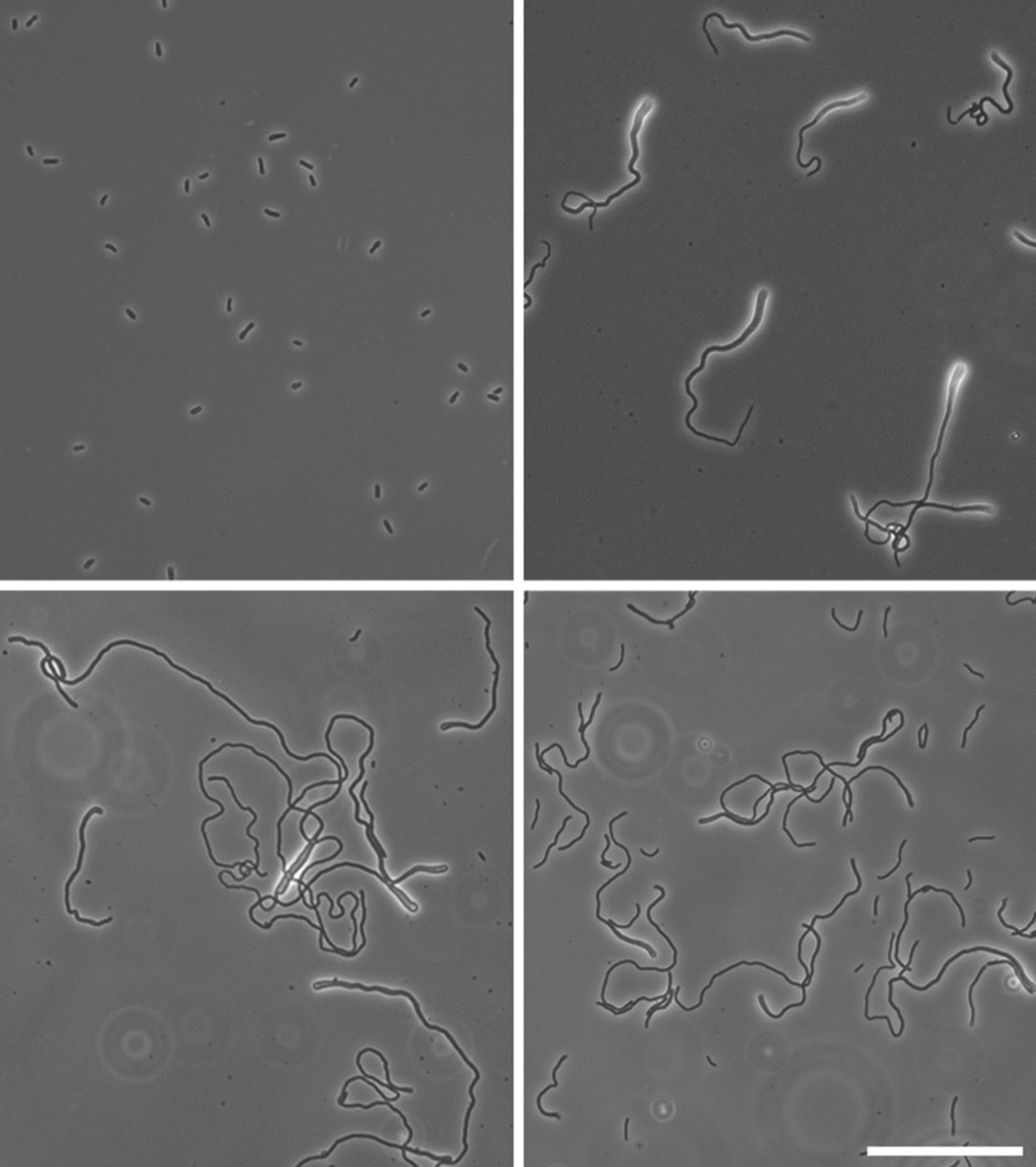
Improved morphology of evolved *Z. mobilis* strains under salt conditions. All images were taken by a phase contrast microscope. Left top; growing Zm6 in the complex medium without addition of salt. Right top; growing Zm6 in the medium containing 0.225 M NaCl, showing abnormal bulged shape at a pole of elongated cells. Left bottom; growing strain KFS1 in the complex medium with 0.225 M NaCl, showing long filamentation of cells with occasional bulged shapes. Right bottom; growing strain KFS2 in the complex medium with 0.225 M NaCl, exhibiting shorter filamentation and less occurrence of bulged cell pole comparing to KFS1. Scale bar applies to all images; 50 μm

**Fig. 3:**
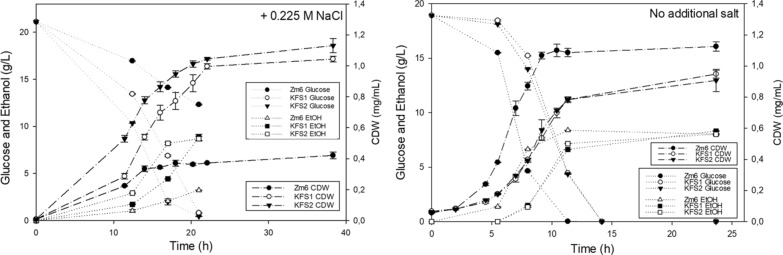
Fermentation profiles of studied strains under salt or non-salt conditions. The plots show growth curve, glucose consumption and ethanol production under the saline (0.225 M NaCl) condition (left panel) or the non-salt condition (right). The error bars represent standard deviations of 3 independent measurements. The strains KFS1 and KFS2 exhibited improved growth and fermentation capacity under the saline condition, while these strains grew mildly slower than the parental strain did under the non-salt condition

Previously, it was shown that salt condition induces filamentation of *Z. mobilis* cells [11]. We observed the filamentation in our experimental setting, using our standard complex medium supplemented with 0.225 M NaCl (Fig. 2). Interestingly, almost all cells exhibited an abnormal bulge at single cell pole, with variant cell length and width (Fig. 2 top right). These cells constituted a loosened pellet, found as sediment in the fully-grown culture (Fig. 1), however, we did not observe any floc formation which was shown to be beneficial for stress resistance in *Z. mobilis* [25]. Based on the cell shapes and the growth profile (Figs. 2, 3), we speculated that the filamentous shape, especially a bulged pole, was a consequence of stress, rather than an adaptation to environment. The similar morphology of bulged filamentation was observed in the *Z. mobilis* cells growing under the high-temperature conditions (39 °C) [26]. We then thought the bulged filamentation to be exploited as a biomarker to identify the stressed cells that should be avoided for serial transfer. Although the sedimentation might have involved other factors, microscopic observations led us to conclude that filamentation of cells facilitated sediment formation.

Following this rationale, we performed lab-directed evolution with a bias in transfer. In practice, we collected cells only from an upper layer in the fully-grown culture as an inoculum for the next round (Fig. 1). The Zm6 strain was adapted in the complex medium supplemented with 0.2 M NaCl for 13 transfers, then continued in the medium with 0.225 M NaCl for another 8 transfers. After the first 10’ about transfers, it was apparent from the turbidity of cultures that the strains demonstrated improved growth characteristics. Strains after the 13th transfer was designated as KFS1 and after the 21st transfer as KFS2. To clarify if the improved growth was due to temporal physiological adaptation without genetic mutation or whether the stable phenotype arose from mutations, we performed a pilot experiment. The evolved strain culture without adaption, i.e., grown in the medium without additional salt, was used as an inoculum for measuring the growth profile under salt condition (0.225 M NaCl). We observed the improved growth, excluding the possibility of transient adaptation and showing that mutation in the genome was the cause.

The evolved strains exhibited two characteristics in cell shape under the salt condition. The strains less frequently formed bulged pole than the parental strain did (Fig. 2, bottom panels), indicating that the evolved cells were less stressed under the salt condition. The evolved cells were also found fragmented or long filament shaped (Fig. 2).

### Characterization of evolved strains

Next, we characterized phenotypes of presumably evolved strains by determining the growth curve, glucose consumption and ethanol production of the evolved and the parental strains in the presence of various salt concentrations. It should be noted that the used inoculum for the culture was not adapted to salt condition. (See Materials and methods).

As shown in the Fig. 3, the parental strain Zm6 did not consume all available glucose in the medium with salt, due to an arrest of growth. In sharp contrast, the evolved strains exhibited improved growth and ethanol production under the salt condition, compared to those by the parental strain under the same condition (Fig. 3). Remarkably, the final biomass of evolved strain was about 2.5 and 2.7 times higher than that of Zm6, respectively for KFS1 and KFS2 (Fig. 3). The total ethanol production by KFS1 and KFS2 was also significantly improved, 2.74 and 2.69 times higher than by the parental strain (Fig. 3). It should be noted that the final biomass (mg.mL^−1^) and the ethanol yield [EtOH(g)/Glucose(g)] by the evolved strains under the salt condition was close to those by the parental strain under the non-salt condition.

**Fig. 4:**
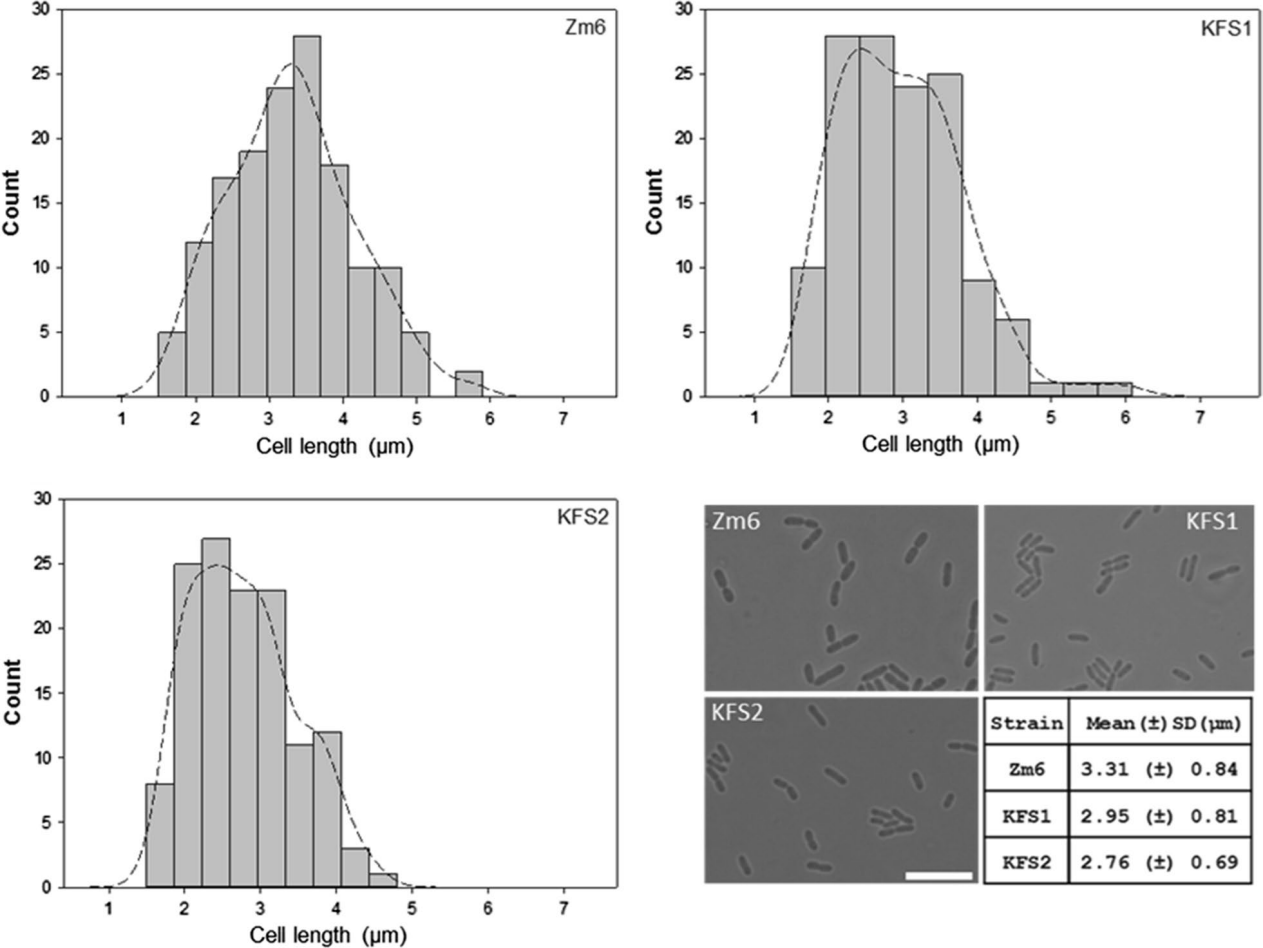
Cell sizes are reduced in the evolved strains under non-saline conditions. Histograms (top two and left bottom panel) show the distribution of cellular length of growing Zm6 (top left), KFS1 (top right) and KFS2 (left bottom) under the non-saline condition (N = 150 for Zm6, N = 133 for KFS1 and KFS2). The cells were grown in the complex medium under anaerobic condition until OD_600_ of the cultures reached around 1 in all strain. Cells were then mounted on the agarose-pad and imaged by a phase contrast microscope. Kernel density was plotted using bandwidth of 0.289 for all histograms. Phase contrast images of each strain under non-saline condition (left bottom, strain is indicated in the image) is shown with a scale bar 10 μm applied for all images. Small table in the right bottom displays the mean value of cellular length (μm) in each strain. SD stands for standard deviation

To characterize the strains further, growth profiles of all strains in the medium without supplement of salt were recorded (Fig. 3). Both evolved strains showed slightly retarded growth and ethanol production under non-salt condition, and their final biomass was significantly lower than that of the parental strain. This was somewhat expected by us, considering that the improvement of growth in salt medium was drastic and likely involved a physiological alternation. However, the final ethanol production by all strains was nearly same (Fig. 3), showing that the final ethanol production per cell dry weight was, interestingly, higher in the evolved strains. This implies that the enzymes responsible for fermentation was more dense in the evolved strains than the parental strain. Along with this line, we observed that the evolved cells exhibited smaller cell size than the parental strain, as shown by light microscopic images (Fig. 4).

### Quantitative metabolomics

The bacterial osmotic response generally involves an accumulation of osmoprotectant to counter an external osmolality, to maintain turgor. Proline and betaine-glycine are examples of well-characterized osmoprotectant in several bacteria [27]. Such a response, accumulating specific compounds as osmolytes, may result in dynamic metabolic changes and could perturb production of desirable compounds by *Z. mobilis*. To determine if any metabolomic adjustments played a significant role in the acquired resilience in the evolved strains, we performed quantitative targeted metabolic profiling of central carbon metabolites and free amino acids in all strains.

**Fig. 5:**
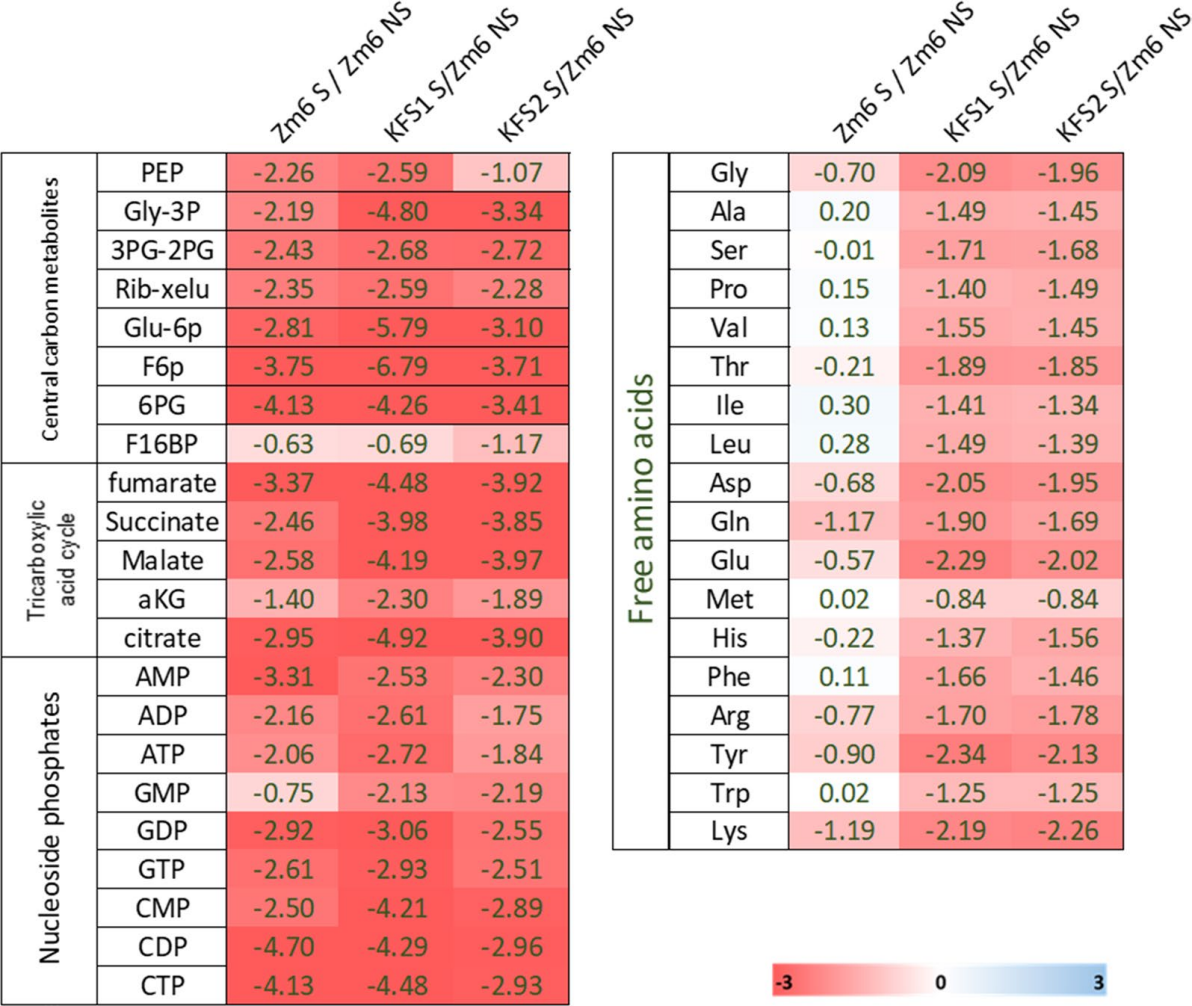
A heatmap representing the log2 fold change of measured metabolites between different conditions or strains. The left column represents the ratio of Zm6 under non-salt condition (Zm6 NS) versus Zm6 under salt condition (Zm6 S). The middle column shows the ratio Zm6 NS versus KFS1 under the salted condition (KFS1 S) and right column shows the ratio Zm6 NS versus KFS2 under the salted condition (KFS2 S). The numbers are log2 fold change of metabolites abundance. A table of absolute abundance (pmol/CDW mg) and abbreviation of metabolites are found in supplementary material (Additional file 1: Figure S1 and Table S1). Colour gradient represents a scale of log2 value. NS in sample designation stands for growth condition without a supplement of salt in medium while S stands for the condition with 0.225 M NaCl in the medium

Our initial goal was to obtain intracellular concentrations for a comparative analysis. The challenge here was that cellular volume was highly heterogeneous in all strains under the salt condition (Fig. 2), hindering the intracellular concentration measurements that require defined cell volume. Therefore, we first normalized metabolite abundance in each strain by its cell dried weight (Fig. 5). The normalized metabolites abundance was compared between strains and conditions, as shown by the heatmap of log2 fold change in the Fig. 5. From fixed weight of cell extracts, Zm6 cells without stress (Zm6 NS) generally comprised larger pools of the metabolites than Zm6 cells under the salt condition (Zm6 S) (Fig. 5, left panel). Interestingly, several free amino acids including proline were the only metabolites found upregulated under the salt condition per fixed dry weight, although mildly. Similarly, the extracts from evolved strains under the salt condition (KFS1 S, KFS2 S) showed smaller pool sizes of the metabolites than from Zm6 NS (Fig. 5, middle and right panel).

**Fig. 6:**
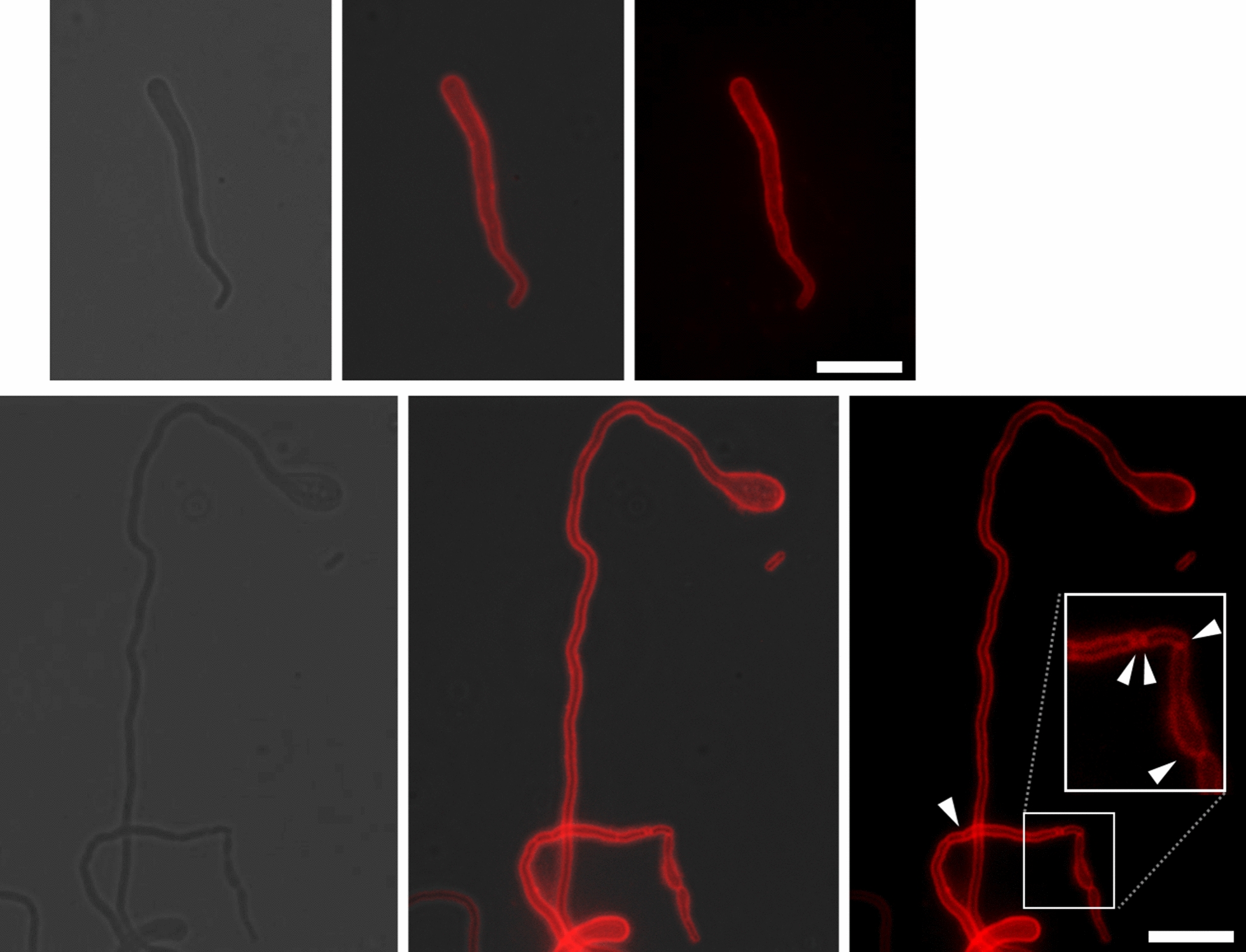
The membrane staining of Zm6 and KFS1 strains growing under the salt condition. The growing Zm6 and KFS1 cells under salt condition (NaCl 0.225 M) was stained by Fm4-64 at concentration of 20 μg.ml^−1^ for 15 min for the membrane visualization. The dye was washed by PBS prior to mounting on the agarose-pad for fluorescent microscopy. Imaging revealed that the bulged Zm6 cell (top panels) did not show any septa nor membrane compartment inside of cells. The KFS1 cell (bottom panels) occasionally formed very long filaments over 50 μm without any septa within cell. Locally frequent septa formation was found in some KFS1 cells, as pointed by white arrowheads. These suggest that septation was not tightly controlled in the strain under the salt condition. Red; Fm4-64 fluorescent signal. Scale bar; 10 μm

Next, we stained the membrane of *Z. mobilis* cells with a staining dye Fm4-64 to observe if there was a compartmentalization within the cell. We observed no septum formation nor membrane organelle in the bulged Zm6 cells (Fig. 6). Apparently, the salted Zm6 single cell volume was much larger than that of rod shape Zm6 cell under non-salt condition (Figs. 2, 6).

According to the previous studies, protein, DNA and RNA are the main components constituting about 70–80% of weight in bacterial cells [28]. It is less likely that slow growing filamentous cells (Zm6 S) possessed more dense macro molecules per fixed cell volume than actively growing small cells (Zm6 NS). We therefore speculated that Zm6 S is expected to have larger cell volume per fixed amount of CDW than that of Zm6 NS. This further leads to that the log2 ratio of Zm6 S/Zm6 NS in the Fig. 5, normalized by CDW, should decrease when intracellular concentration is deployed as a unit, as Zm6 S cells most likely possessed larger cell volume per same amount of CDW, in comparison to Zm6 NS cells. Thus, our metabolomics data suggest that Zm6 cells did not drastically accumulate central metabolites or free amino acids under the salt condition. Most of the ED pathway metabolites and nucleosides in Zm6 S was drastically downregulated, coinciding with low growth and slow glucose uptake. Moreover, osmoprotectant such as proline, was not found significantly accumulated either. Although, it was not completely excluded that atypical osmoprotectants that were not included in our targeted analysis might have been upregulated in Zm6 S cells during the stress response.

The KFS1 and KFS2 cells under saline condition exhibited heterogeneous cell shapes, ranging from fragmented cells to long extended cells (Fig. 2). The membrane staining showed that the cells could produce a septum at several locations, unlike Zm6 cells. Nevertheless, KFS1 cells did not complete division and instead formed long filaments, and KFS1 S cell compartment size was found overall larger than that of Zm6 S (Fig. 6). Similar to the case of comparison of Zm6 extracts between saline conditions, bigger volume and slower growth of KFS1 S and KFS2 S to Zm6 NS implied that the actual intracellular concentration ratio of KFS1 or KFS2 S to Zm6 NS was smaller than the log2 ratio depicted in the Fig. 5. This suggests that the evolved strains did not accumulate osmolytes to counter the stress either.

To further understand the resilient mechanism in evolved strains, we measured the intracellular ratio of reduced/oxidized form of NAD cofactor (NADH/NAD) by an enzymatic assay. Malate dehydrogenase and oxoglutarate dehydrogenase complex are not encoded in the *Z. mobilis* genome [3, 29], which influences regeneration of NAD. Previous work suggested that maintaining a low NADH/NAD ratio appear to be important in the response against salt and acetic acid stress, to sustain glycolysis in the ED pathway that requires oxidized NAD [15, 30]. To examine if the redox regulation of co-factor conferred the resilience, we examined the NADH/NAD ratio in the strains. The analysis showed that saline stress increased the ratio in Zm6 (Table 1), as it was previously shown [15]. Interestingly, upregulation of the NADH/NAD ratio under saline conditions was also observed in evolved strains as well, within a similar range of shift to Zm6. These data indicate that a modulation of NADH/NAD did not appear to play a major role in the evolved strains. Although the modulation of ratio is vital in general *Z. mobilis* stress response, as shown by previous studies [15, 30], the resilience to salt stress in the evolved appeared to be mediated by a separate mechanism.

**Table 1:** The NADH/NAD ratio in growing Zm6 and evolved strains under the non-saline or the saline conditions

Strain	NADH/NAD (±) SD	
	Non-salt condition	Salt condition
Zm6	0.57 (±) 0.03	0.99 (±) 0.11
KFS1	0.40 (±) 0.05	0.97 (±) 0.20
KFS2	0.51 (±) 0.15	1.08 (±) 0.15

### Identification of gene loci

We examined the genomes of the evolved strains in an attempt to identify mutational changes that might be responsible for the observed stress tolerance phenotype. The whole genomes of parental and evolved strains were sequenced and aligned against the reference genome [29]. The analysis showed that our lab stock Zm6 strain possesses several mutations, 5 point-mutations and 2 frame shift-mutations of the ORFs in its genome (Additional file 1: Table S2). These mutations likely arose during our previous laboratory practices. We found several mutations only arose in the evolved strains (Table 2). A disruptive insertion in ZZ6_1449 coding carboxyl-terminal protease (CTP) was among them. CTP is found in all kingdom of life and mainly cleaves serine or lysine nearby at C-terminus of substrate. In bacteria, it has been shown that mutation in CTP caused alternation in cell envelop and higher sensitivity to antibiotics [31] and the osmotic down-shift in *Escherichia coli* [32]. In *Pseudomonas aeruginosa,* the disruption of CTP resulted in impaired growth in the medium with low salt [33]. Although, it was not clear if the observed phenotypes of low salt sensitivity in *E. coli* and *P. aeruginosa* were a direct consequence of an inactivation of the effector proteins of CTP that are involved in the osmo-protection, or, might be an indirect consequence of the altered PG metabolism caused by the disruption of CTP in the mutant [32, 33]. The reported phenotypes in *E. coli* and *P. aeruginosa* are to some extent consistent with our results from the growth profile. In addition, we found three other point-mutations only found in KFS1 and KFS2, which might be linked to the salt-resilient phenotypes. However, the function of these genes is not annotated. We found a point-mutation only found in KFS2, and an annotation of mutated gene is not available from the database. There are mild differences between KFS1 S and KFS2 S in morphology and growth, and it cannot be excluded that the mutation in the uncharacterized gene conferred extra resilience in KFS2 as well. Together with available literature, it suggests that disruption of ZZ6_1449 was mainly responsible for the improvement of growth. However, the genetic complementation test on the evolved strains, or, an introduction of disruptive mutation to *ctp* locus in the parental Zm6 strain should be performed to prove the link between the CTP mutation and the improved phenotype.

**Table 2:** A list of mutations only found in the evolved strains

Position	Detected sequence	Locus	Mutation	(annotated) Function	Strains
7181	C -> T	ZZ6_0006	A273T	Carboxymethylenebutenolidase	KFS1 and KFS2
342703	C -> T	ZZ6_0303	G192R	Hypothetical protein	KFS1 and KFS2
1641047	A -> AGGCTCAGGACCCATTGATTT	ZZ6_1449	Insertion at L282, frame shift	Carboxyl-terminal protease	KFS1 and KFS2
1650537	C -> T	ZZ6_1458	G139V	Hypothetical protein	KFS2

## Conclusion

The present study successfully generated strains that can grow and produce ethanol in added salt media usually inhibitory to wild type strains. Our approach was to bias selection by sedimentation of stressed filamentous cells, which might be utilized in other kinds of strain improvement in *Z. mobilis*. Interestingly, the evolved strains did not adapt to the saline environment by adjusting the prototypical osmolyte concentration or modulation of the intracellular NADH/NAD ratio. Comparative genome sequencing of wild type and evolved strains revealed that the disruption of *ctp* was likely responsible for the improvement by altering cell envelope profile, yet an experimental evidence is required for confirming the disruption of CTP as a cause of the phenotype in the evolved strains. Further elucidation of the evolved strains might shed light on mechanistic understanding of salt stress response in *Z. mobilis*.

## Materials and method

### Adaptive laboratory evolution

*Z. mobilis* ATCC 29191 was used as a parental strain for laboratory evolution. Zm6 was cultivated in a complex growth medium containing glucose (20 g/L), yeast extract (5 g/L), NH_4_SO_4_ (1 g/L), KH_2_PO_4_ (1 g/L), MgSO_4_ (0.5 g/L), with supplemental NaCl (final concentration; 0.2 M–0.225 M) to generate a salt tolerant strain. The complex medium was flashed by nitrogen gas filtered through sterilized 0.2 μm Supor^®^ (polyethersulfone) membrane (PALL) prior to use. A glycerol stock from a −80 °C-freezer was inoculated into the medium to make a starter culture. It should be noted that the glycerol stock was made from the overnight culture originated from a single colony of our Zm6 lab stock strain. 12 mL of anaerobic Zm6 culture was grown at 30 °C in a tightly capped 15 ml falcon tube with shaking at 200 rpm. 30 μL of inoculum was transferred from previous round of culture for evolution. Transfer of cells to fresh medium was performed when cells reached stationary phase. First 13 transfers were done in the medium with 0.2 M salt, generating KFS1 strain. The rest of evolution was performed in the medium with 0.225 M for another 8 transfers, generating KFS2 strain.

### Characterization of evolved strains

To analyze cellular growth under the salt condition, an overnight culture was inoculated into anaerobic standing cultures and incubated at 30 °C. It should be noted that the used inoculum was grown in the same growth medium without additional NaCl, thus the inoculated cells were not adapted to the salt condition. The optical density (absorbance at 600 nm) of culture was measured using a spectrophotometer (VWR). The cell dry weight was measured as follows; growing cells were centrifuged for 5 min (4500*×g*) and washed in phosphate buffered saline (PBS) solution (OXOID). The washed cells were transferred to a foil cup package (VWR) and dried in an oven at 90 °C till the solutions got completely dried. The dried cells on foil were weighed and subtracted by the tare weight of foil and dried PBS. Figure 3 was made using the measured OD_600_ and the conversion factors CDW [g/L]/OD_600_ (Additional file 1: Table S3).

Determination of glucose consumption and ethanol production in spent media was performed using Waters 2695e Alliance HPLC (Waters) with Hi-plex column (300 × 7.7 mm, Agilent) under a running condition; 0.05 M sulfuric acid as mobile phase at a flow rate of 0.8 mL/min. MQuant^®^ Glucose test kit (Merck) was also tested for an estimation of glucose presence (10–500 mg/L).

### Metabolomics

Cell extractions were prepared as described in [34, 35]. Central carbon metabolites and nucleoside-phosphate were analyzed by capillary ion chromatography [34] coupled to tandem mass spectrometry, TQ-XS (Waters). Free amino acids were first derivatized by Edman’s reagent [36]. Derivatized samples were analyzed by UPLC (waters) coupled to TQ-XS as described in [37]. All metabolites measurements were corrected by isotopic dilution method as described in [37].

Determination of the NADH/NAD ratio was performed using an enzymatic assay kit, following the instruction supplied by the manufacturer (Sigma). Briefly, growing culture was centrifuged (3 min, 4500×*g*) and the supernatant was carefully discarded. Pellets were quickly frozen by liquid nitrogen for storage at −80 °C. Upon the measurements, the frozen cells were thawed on ice and resuspended in the extraction buffer supplied from the kit. An absorbance at 450 nm was measured every 20 min for 4 h using 96 well-plates in a plate-reader spark 20 M (Tecan).

### Fluorescent microscopy and image analysis

Live *Z. mobilis* cells were observed using a Zeiss Axio Imager Z2 microscope. The images were captured by Axiocam MR R3 (ZEISS) and analyzed by ZEN 2.3 pro software (ZEISS). For staining membrane, growing cultures were centrifuged (4500x*g*) for 5 min and the pellet was resuspended in phosphate buffered saline solution. FM4-64 (Thermo Fisher Scientific) was then added at a final concentration of (20 μg.ml^−1^) and incubated for 15 min. Cells were then washed in PBS again and mounted on PBS agarose-pad (1% w/v). Comparison of cell size was done using the phase contrast images and the scale bars obtained from the imaging.

### Whole genome sequencing

The whole genome of the studied strains was sequenced by GATC re-sequencing service (INVIEW Genome sequencing). Total DNA extraction was performed combining a lysing method [38] and the D-neasy blood tissue kit (Qiagen). Briefly, a lysozyme treatment was performed as follows. An overnight culture was centrifuged (20,000x*g*) for 1 min, and the pellets were washed in TE buffer (10 mM Tris–Cl pH7.5, 1 mM EDTA) and centrifuged again. The pellets were resuspended in a lysozyme solution and incubated for 30 min at 37 °C. (Lysozyme solution; lysozyme 50 mg.ml^−1^ in buffer containing 10 mM Tris–Cl pH 8, 2.5 mM EDTA, 20 mM NaCl). This procedure was followed by a proteinase K incubation (20 mg.ml^−1^, 1 h at 50 °C) and the rest of procedures followed the instructions provided by the manufacturer. A concentration and purity of genomic DNA was determined using nanodrop one (thermo scientific).

## Supplementary information

**Additional file 1: Figure S1.** Metabolite abundance [pmol.mg^−1^ (CDW)] in each strain was measured by mass spectrometry-based metabolomics. The data was used for log2 fold ratio heatmap in the Fig. 5. **Table S1**. A list of abbreviation of measured metabolites. **Table S2**. A list of detected mutations in the lab stock Zm6 strain. **Table S3**. The CDW/OD_600_ conversion factor in each strain under saline/non-saline conditions.

## Data Availability

The datasets and strains generated from the present study are available from the corresponding author on a reasonable request.

## Abbreviations

ED: Entner-Doudoroff
NAD: Nicotinamide adenine dinucleotide
CDW: Cell dry weight
OD: Optical density
HPLC: High pressure liquid chromatography
UPLC: Ultra-performance liquid chromatography
SD: Standard deviation
WGS: Whole genome sequencing
