# Supplementary material for: Increased salt tolerance in Zymomonas mobilis strain generated by adaptative evolution
Source: Microb Cell Fact. 2020 Jul 20;19:147. doi: 10.1186/s12934-020-01406-0 (PMC7372843; doi:10.1186/s12934-020-01406-0)

Figure S1. Metabolite abundance [pmol.mg^-1^ (CDW)] in each strain was measured by mass spectrometry-based metabolomics. The data was used for log2 fold ratio heatmap in the Figure 5.


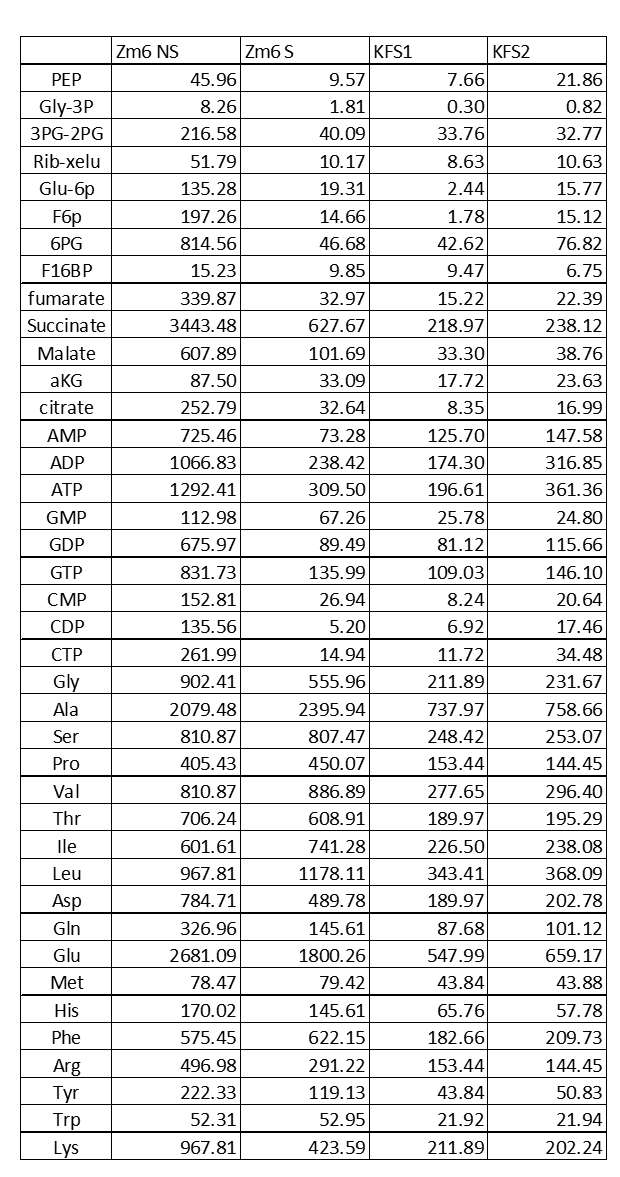


Table S1. A list of abbreviation of measured metabolites.


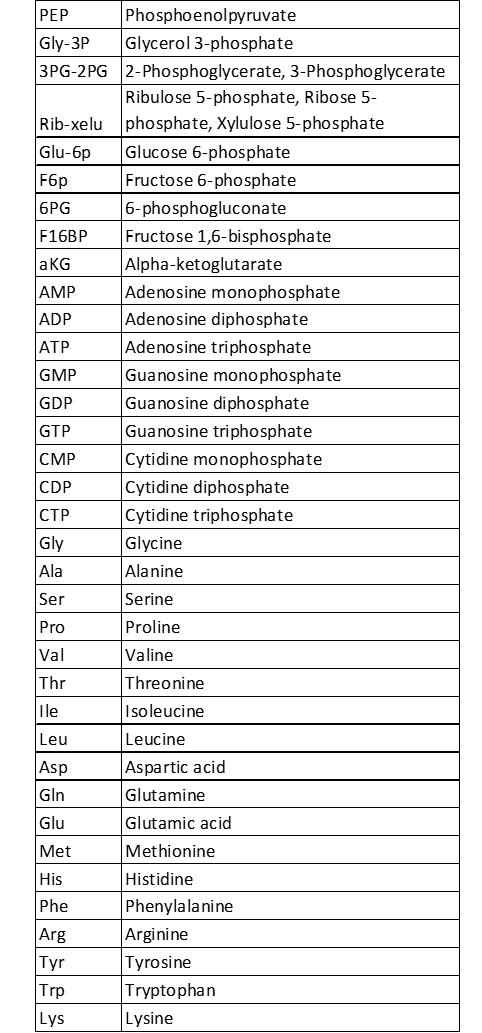


Table S2. A list of detected mutations in the lab stock Zm6 strain.


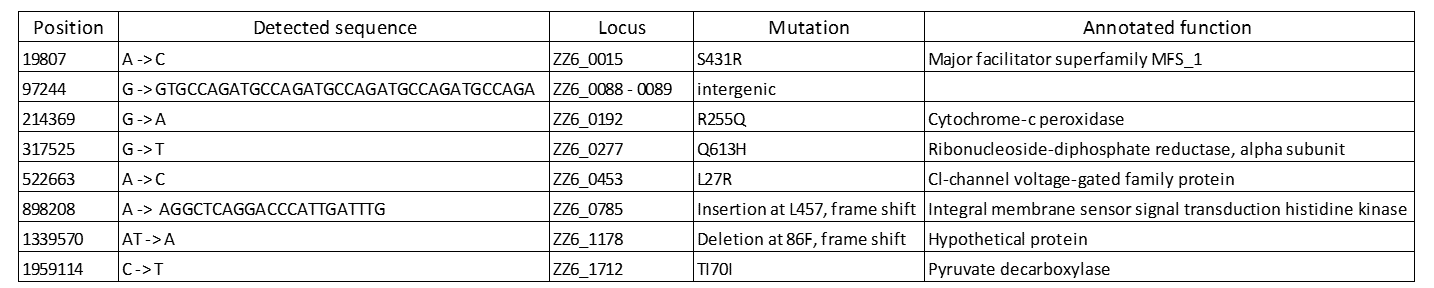


Table S.3 The CDW/OD_600_ conversion factor in each strain under saline/non-saline conditions.


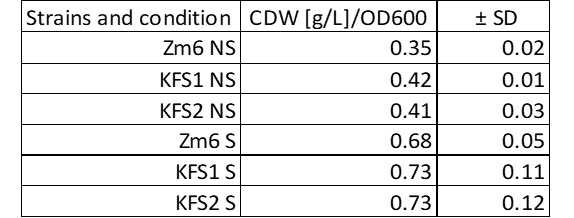

Supplement: Supplementary file 1 — Additional file 1: Figure S1. Metabolite abundance [pmol.mg−1 (CDW)] in each strain was measured by mass spectrometry-based metabolomics. The data was used for log2 fold ratio heatmap in the Fig. 5. Table S1. A list of abbreviation of measured metabolites. Table S2. A list of detected mutations in the lab stock Zm6 strain. Table S3. The CDW/OD600 conversion factor in each strain under saline/non-saline conditions. [file 12934_2020_1406_MOESM1_ESM.docx]
